# Validation of a Novel Device for the Knee Monitoring of Orthopaedic Patients

**DOI:** 10.3390/s19235193

**Published:** 2019-11-27

**Authors:** Mahmut Enes Kayaalp, Alison N. Agres, Jan Reichmann, Maxim Bashkuev, Georg N. Duda, Roland Becker

**Affiliations:** 1 Department of Orthopaedics and Traumatology, Brandenburg Medical School Theodor Fontane, 14770 Brandenburg, Germany; mek@mek.md; 2 Istanbul University-Cerrahpasa Cerrahpasa, Faculty of Medicine, 34098 Istanbul, Turkey; 3 Julius Wolff Institute, Charité-Universitätsmedizin Berlin, 13353 Berlin, Germany; alison.agres@charite.de (A.N.A.); maxim.bashkuev@statconsult.de (M.B.); georg.duda@charite.de (G.N.D.); 4 StatConsult GmbH, 39112 Magdeburg, Germany; jan.reichmann@statconsult.de

**Keywords:** Telemedicine, remote monitoring, fast-track surgery, knee activity monitor, inertial sensors

## Abstract

Fast-track surgery is becoming increasingly popular, whereas the monitoring of postoperative rehabilitation remains a matter of considerable debate. The aim of this study was to validate a newly developed wearable system intended to monitor knee function and mobility. A sensor system with a nine-degree-of-freedom (DOF) inertial measurement unit (IMU) was developed. Thirteen healthy volunteers performed five 10-meter walking trials with simultaneous sensor and motion capture data collection. The obtained kinematic waveforms were analysed using root mean square error (RMSE) and correlation coefficient (CC) calculations. The Bland–Altman method was used for the agreement of discrete parameters consisting of peak knee angles between systems. To test the reliability, 10 other subjects with sensors walked a track of 10 metres on two consecutive days. The Pearson CC was excellent for the walking data set between both systems (r = 0.96) and very good (r = 0.95) within the sensor system. The RMSE during walking was 5.17° between systems and 6.82° within sensor measurements. No significant differences were detected between the mean values observed, except for the extension angle during the stance phase (E1). Similar results were obtained for the repeatability test. Intra-class correlation coefficients (ICCs) between systems were excellent for the flexion angle during the swing phase (F1); good for the flexion angle during the stance phase (F2) and the re-extension angle, which was calculated by subtracting the extension angle at swing phase (E2) from F2; and moderate for the extension angle during the stance phase (E1), E2 and the range of motion (ROM). ICCs within the sensor measurements were good for the ROM, F2 and re-extension, and moderate for F1, E1 and E2. The study shows that the novel sensor system can record sagittal knee kinematics during walking in healthy subjects comparable to those of a motion capture system.

## 1. Introduction

Rehabilitation is an important complement to both conservative and surgical treatment of orthopaedic patients. With the increased trend towards fast-track surgery [1], its importance has increased even further. Therefore, technological supplements like telemedicine are expected to gain popularity [2], especially in rural areas, where patients may have less contact with their orthopaedic surgeons.

Total knee arthroplasty (TKA) provides a significant improvement in the quality of life for knee osteoarthritis patients [3,4,5]. As life expectancy and activity levels in the elderly increase in industrialised countries, TKA is becoming even more popular [6]. Aftercare of these patients in the postoperative period is an area of interest. Current practices of fast-track surgery and postoperative home-based exercise protocols necessitate remote monitoring of patients to objectively evaluate the rehabilitation process. Therefore, remote monitoring devices capable of objectively measuring knee kinematics representative of physical activity are of interest. The objective and qualitative assessment of patients’ performance after surgical treatment with TKA would help to characterise the changes in physical function and mobility of patients [7]. One of the key factors for a fast and successful recovery is the level of the patient’s physical activity [6], but no appropriate tools for its objective assessment in the postoperative period are currently available [8]. Telerehabilitation has been shown to be successful and beneficial for both patients and clinicians in providing a more objective clinical outcome assessment [9]. The continuous monitoring of patients’ mobility, and specifically knee motion, would moreover allow for a better assessment of the success of therapy and for an early detection of potential functional disabilities, such as a lack of expected range of motion and extent of mobility in terms of exercise monitoring. It could also guide patients through the rehabilitation programme and help clinicians optimise the programme by providing information about the intensity and frequency of exercises and general patient compliance, and thus contribute to patient satisfaction [9].

**Figure 1:**
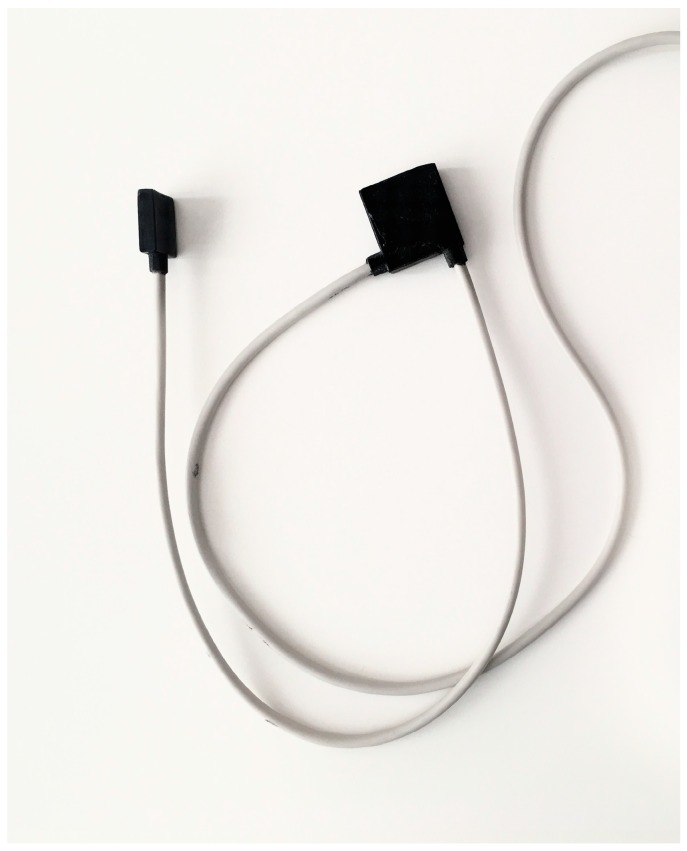
The system was comprised of two sensor units, one of which was larger and acted as a master unit synchronising the sensor data. The units were connected via a cable.

This motivated the development of a wearable system for the monitoring of knee functions and mobility (Figure 1). The system is based on inertial sensors. It has an advantage over laboratory methods for motion analysis, like camera-based motion capture or videofluoroscopy. The advantage is that it allows for the assessment of knee motion in a field setup without restricting patients in their daily life. However, because attachment of the sensors to the upper and the lower leg can be arbitrary and there is no practical way to ensure that the sensor axes coincide with the physiological joint axis of the knee, a complex system calibration is necessary for a reliable assessment of knee motion [10]. While preliminary validation attempts on a robotic arm (KUKA Youbot, KUKA AG, Augsburg, Germany) demonstrated good precision and repeatability of measurements (unpublished internal data), the performance of the system on a living subject has yet to be confirmed.

Therefore, the present study aimed to validate the developed system by comparing it to motion capture, a gold standard method in gait analysis, and evaluate its applicability in the monitoring of sagittal knee kinematics.

## 2. Material and Methods

### 2.1. The Sensor System

The system comprised of two sensor units, each including a nine-degree-of-freedom (DOF) inertial measurement unit (IMU) (BNO055, Bosch Sensortec GmbH, Reutlingen, Germany). In the evaluated prototype, the sensors (unit 1: 2.4 cm × 2.4 cm × 0.4 cm, unit 2: 1.8 cm × 1.8 cm × 0.4 cm) were connected via cable to one of the sensors acting as a “master” synchronising the sensor data (Figure 1). This sensor, in turn, was connected to a storage unit (size: 12.5 cm × 5.5 cm; mass: 80 g), which transmitted the synchronised sensor data to a tablet with a custom-developed app to remotely control the measurement process and calculate the knee angle. After the measurements, the data were downloaded from the tablet via a USB. The chip of the developed IMU system contained an implemented magnetic rejection algorithm, which switched the device to a 6-DOF mode if a strong magnetic distortion was detected, and switched back to a 9-DOF mode when the distortion was no longer present. 

### 2.2. Sensor-to-Segment Calibration

The aim of the system calibration was to determine the placement of the sensors relative to the knee joint axis based on a set of arbitrary motions. Assuming the knee is a hinge joint and the sensors are rigidly attached to each of the segments, one can see that the unit vectors $\vec{j_{1}}$ and $\vec{j_{2}}$ defining the joint axis $\vec{j}$ in the local coordinate system of the corresponding sensors remain constant, regardless of joint position. Moreover, while the orientation of each segment is a function of time, the dot product is time-invariant:

(1) $$\left[q_{1}\left(t\right)\vec{j_{1}}q_{1}{}^{-1}\left(t\right)]\cdot [q_{2}\left(t\right)\vec{j_{2}}q_{2}{}^{-1}\left(t\right)\right]=1.$$

Here, $q_{i}\left(t\right)\vec{j_{i}}q_{i}{}^{-1}\left(t\right)$ is the rotation of the local unit vector $\vec{j_{i}}$ by the orientation quaternion $q_{i}$ of the corresponding sensor at time point *t*. This means that the function:

(2) $$r\left(\vec{j_{1}},\vec{j_{2}}\right)=1-\left[q_{1}\vec{j_{1}}q_{1}{}^{-1}]\cdot [q_{2}\vec{j_{2}}q_{2}{}^{-1}\right]$$

should evaluate to be zero at any position of the joint, i.e., for any set of sensor data $\left(q_{1},q_{2}\right)$. Since in reality, the knee is not an ideal hinge joint and the sensors are attached to the skin, skin motion and soft tissue deformation during movement make it impossible to find a pair of vectors ${\vec{j}}_{1}$ and ${\vec{j}}_{2}$ that satisfy the condition for all $\left(q_{1},q_{2}\right)$. However, having a sufficiently large set of sensor data, a least-squares solution of Equation (1) can be found, which is the solution of the following optimisation problem:

(3) $$\underset{x\in {\mathbb{R}}^{4}}{min}\sum_{i=1}^{n}r_{i}^{2}\left(x\right),$$

where $x={\left(\varphi_{1},\theta_{1},\varphi_{2},\theta_{2}\right)}^{T}$ contains spherical coordinates of the axes such as:

(4) $$\begin{array}{c} j_{1}={\left(\cos\left(\varphi_{1}\right)\cos\left(\theta_{1}\right),\cos\left(\varphi_{1}\right)\sin\left(\theta_{1}\right),\sin\left(\varphi_{1}\right)\right)}^{T},\\ j_{2}={\left(\cos\left(\varphi_{2}\right)\cos\left(\theta_{2}\right),\cos\left(\varphi_{2}\right)\sin\left(\theta_{2}\right),\sin\left(\varphi_{2}\right)\right)}^{T}, \end{array}$$

and $r_{i}\left(x\right)$ corresponds to Equation (2) evaluated at the ith element of the data set. Similarly to the previously published algorithm [10], a Gauss–Newton algorithm was implemented. The *i*th row of the Jacobian matrix $J_{r}=\frac{d}{dx}r\left(x\right)$ can be calculated using $\frac{dr}{dj}{|}_{i}\cdot \frac{dj}{dx}$, where $\frac{dr}{dj}{|}_{i}=-\left({\left(q_{1}{}^{-1}\vec{j_{2}}q_{1}\right)}^{T},{\left(q_{2}{}^{-1}\vec{j_{1}}q_{2}\right)}^{T}\right)$ is a 1 × 6 row vector corresponding to the *i*th element of the data set and

(5) $$\frac{dj}{dx}=\left(\begin{array}{cc} \frac{\partial j_{1}}{\partial x} & 0\\ 0 & \frac{\partial j_{2}}{\partial x} \end{array}\right)\mathrm{with}\frac{\partial j_{i}}{\partial x}=\left(\begin{array}{cc} -\sin\left(\varphi_{i}\right)\cos\left(\theta_{i}\right) & -\cos\left(\varphi_{i}\right)\sin\left(\theta_{i}\right)\\ -\sin\left(\varphi_{i}\right)\sin\left(\theta_{i}\right) & \cos\left(\varphi_{i}\right)\cos\left(\theta_{i}\right)\\ \cos\left(\varphi_{i}\right) & 0 \end{array}\right)$$

can be calculated once per iteration step. In each iteration *s*, the next approximation is determined to be $x^{\left(s+1\right)}=x^{\left(s\right)}-J_{r}^{-1}r\left(x^{\left(s\right)}\right)$, with $J_{r}^{-1}$ being the pseudo-inverse of the Jacobian matrix. The solution converges to one of the two possible candidates, which differ only by the sign determining the motion direction. The correct plane of the calculated knee motion can either be determined during the measurement or predefined by prescribing approximate orientations of the sensor attachment.

### 2.3. Measurement Protocol

The validation study was conducted after obtaining the local ethics committee approval from the Ethics Committee of Brandenburg (AS 928bB)/2018). Following a power analysis using data from a similar study [11], it was decided to include at least nine subjects to avoid a type I error with α = 0.05 and a type 2 error with β = 0.85. Thirteen healthy subjects (4 females; age: 32.7 ± 3, height: 1.75 ± 0.1 m, body mass: 73.3 ± 16.7 kg, body mass index: 23.5 ± 3.2 kg/m^2^) participated in the study. All participants provided written informed consent prior to entering the study. 

**Figure 2:**
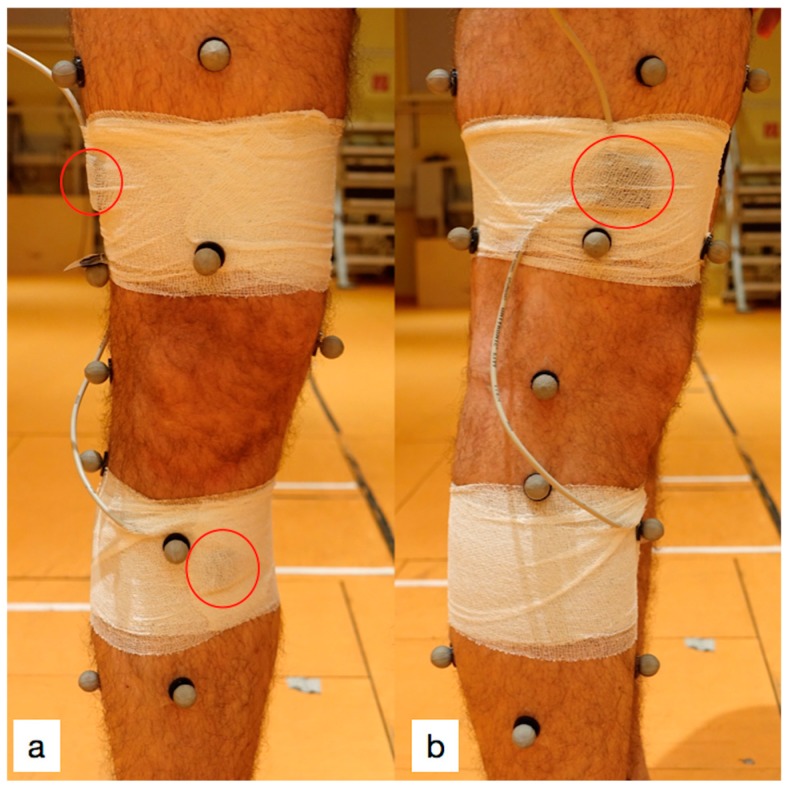
(**a**) Frontal view of the sensor placement. (**b**) Lateral view of the sensor placement. Red circles indicate sensor units. The master sensor unit was placed on the distal lateral femur, 10 cm cranial to the lateral femoral condyle to eliminate disturbances related to soft tissues and muscle contractions. The second unit was placed 5 cm caudal to the knee joint line on the anteromedial aspect of the tibia.

A 10-camera motion capture system (VICON-MX-S, Vicon Motion System LTD, Oxford, UK) collected kinematic data from 31 reflective markers, which were placed on anatomical landmarks as suggested for the OCST, SARA and SCoRE combined approach (OSSCA) [12]. Markers were placed on the following landmarks: anterior and posterior iliac spines, greater trochanter, tibial tuberosity, fibular head, calcaneus, first and fifth metatarsals, medial and lateral femoral epicondyles and ankle malleoli. The remaining markers were optimally placed on the limb [13] for functional identification of the hip joint centre [14] and the knee axes [15]. Participants were barefoot and wore tight shorts for all trials. The two sensor units were fixed above and below the right knee using elastic adhesive bandages (Figure 2). The proximal sensor was placed on the distal lateral femur, 10 cm cranial to the lateral femoral condyle, and the distal sensor was placed 5 cm caudal to the knee joint line on the anteromedial aspect of the tibia. 

Sensor calibration movements consisted of arbitrary movements of the hip and knee joints along different axes. These movements were hip and knee flexions and internal and external hip rotations. There were no minimum or maximum angle limits for these movements. The subjects were then asked to walk along a 10-metre marked pathway in a laboratory setting, turning, then returning to the starting point. Each subject completed this task five times. At both ends of the track, subjects were asked to maximally flex the knee before walking in order to indicate the beginning of the test. Recording frequencies were 50 Hz and 100 Hz for the sensor and motion capture systems, respectively. 

Test–retest reliability was evaluated on ten subjects in a room outside the motion analysis laboratory with 10 metres of available space. These subjects (3 female; age: 42.5 ± 13, height: 1.78 ± 0.12 m, body mass: 76 ± 11.3 kg, body mass index: 23.9 ± 3.35 kg/m^2^) were not included in the validation study. Subjects walked a track of 10 metres in both directions on two consecutive days at a rate of 90 steps per minute using a metronome.

### 2.4. Data Analysis

Kinematic data collected from the motion capture system were post-processed (VICON Nexus 1.8.2, Oxford, UK). Resultant marker trajectories were used to calculate sagittal knee angles with the OSSCA projection [12] using custom scripts written in the MATLAB environment (R2011b, Mathworks, Natick, MA, USA) with reference to a static trial in a neutral position.

**Figure 3:**
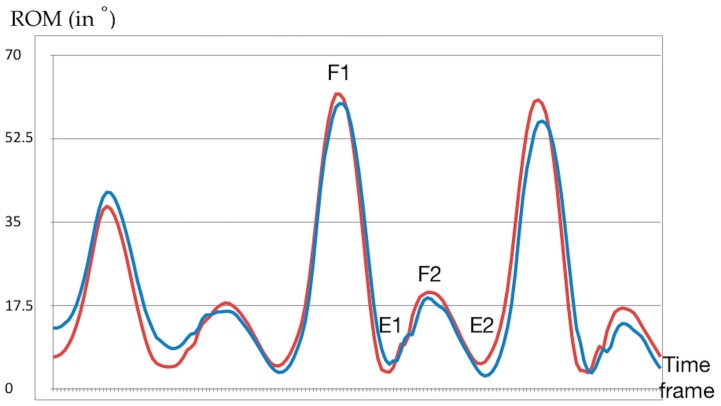
Sample kinematic waveform during walking for a single subject. Discrete parameters used in data analysis are marked as follows: flexion angle in the swing phase (F1), extension angle in the stance phase (E1), flexion angle in the stance phase (F2), and extension angle in the swing phase (E2). Re-extension angle was calculated by subtracting E2 from F2. Blue line: sensor, red line: motion capture.

Calculated knee angles from the motion capture were downsampled to 50 Hz to match the sensor waveform data. Synchronisation of the recorded data from both systems was done using the time point of the peak flexion angle during hyperflexion at the beginning of each trial. Offset calibration [11,16,17] was done using the second extension angle (E2) during the stance phase (Figure 3). All recorded data were cut into single walking strides. Discrete parameters were determined as the peak knee angles, comprising the flexion angle during the swing phase (F1), the flexion angle during the stance phase (F2), the extension angle during the stance phase (E1) and the extension angle during the swing phase (E2) (Figure 3). Additionally, the difference between E2 and F2 was calculated to determine the re-extension angle of the knee during walking (Re-ext) [18,19].

All collected data were analysed (a) between the two systems (n = 13) and (b) for the repeatability assessment (n = 10).

### 2.5. Statistics

All data in the form of kinematic waveforms were analysed by calculating: (1) the Pearson correlation coefficients (PCCs) and associated *p*-values and (2) the root-mean-square error (RMSE). Correlations of kinematic waveforms were evaluated by adding all acquired data in tandem. RMSE values were calculated for each subject, and subsequently, all data were used to determine the mean values. The correlation was evaluated as excellent (>0.95), very good (0.85–0.95), good (0.75–0.85), moderate (0.65–0.75) or weak (<0.65) [11].

The mean range of motion (ROM), as well as the discrete parameters’ mean values, were calculated and analysed. Parameters calculated from the sensors were benchmarked against those calculated from motion capture measurements using a one-sample *t*-test. A Pearson correlation test was conducted for single values of each discrete parameter as recorded by both systems. 

Intra-class correlation coefficients (ICCs) were calculated with a 95% limitation of agreement (1.96 × standard deviation of the difference between the two systems). ICCs were rated as either excellent (0.9–1), good (0.74–0.89), moderate (0.4–0.73) or poor (0–0.39) [11]. Analysis of the agreement between the systems was performed using the Bland–Altman method.

## 3. Results

A post-hoc power analysis was run using the G*Power 3 program. To avoid a type I error with α=0.05, thirteen subjects provided a power of 81.3%. The Pearson correlation coefficient, associated *p*-values and RMSE values are depicted in Table 1. The correlation was excellent between systems (r = 0.96) and very good (r = 0.95) within the sensor system. The RMSE was 5.17° between systems and 6.82° within the sensor measurements.

A one sample *t*-test revealed no significant differences between the observed mean values of parameters, except for E1 (*p* < 0.05) during walking (Table 2). Similar results were obtained for the repeatability test (Table 3).

The ICCs between systems are displayed in Table 4. They were rated as excellent for F1, good for F2 and re-extension, and moderate for E1, E2 and ROM mean values during walking. ICCs within the sensor measurements are presented in Table 5. The ROM, F2 and re-extension mean values had good correlations, and F1, E1 and E2 mean values had moderate correlations.

**Figure 4:**
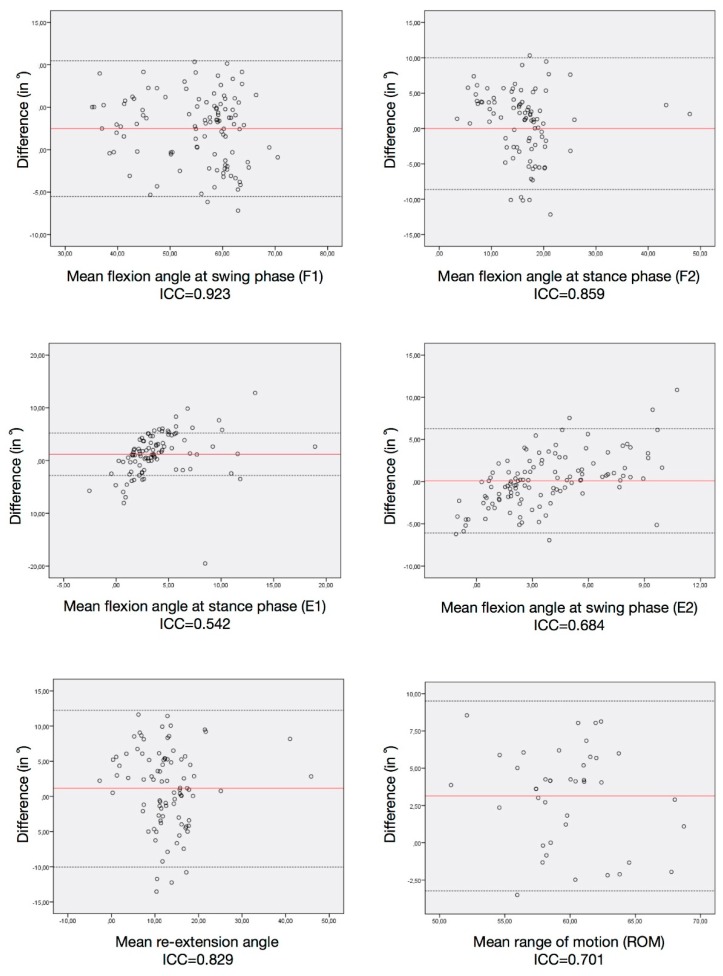
Bland–Altman plots of the discrete parameters from both systems. Each graph represents the mean difference (black line) and 1.96× standard deviation of the difference (dashed lines) as recorded by the sensors and motion capture.

**Figure 5:**
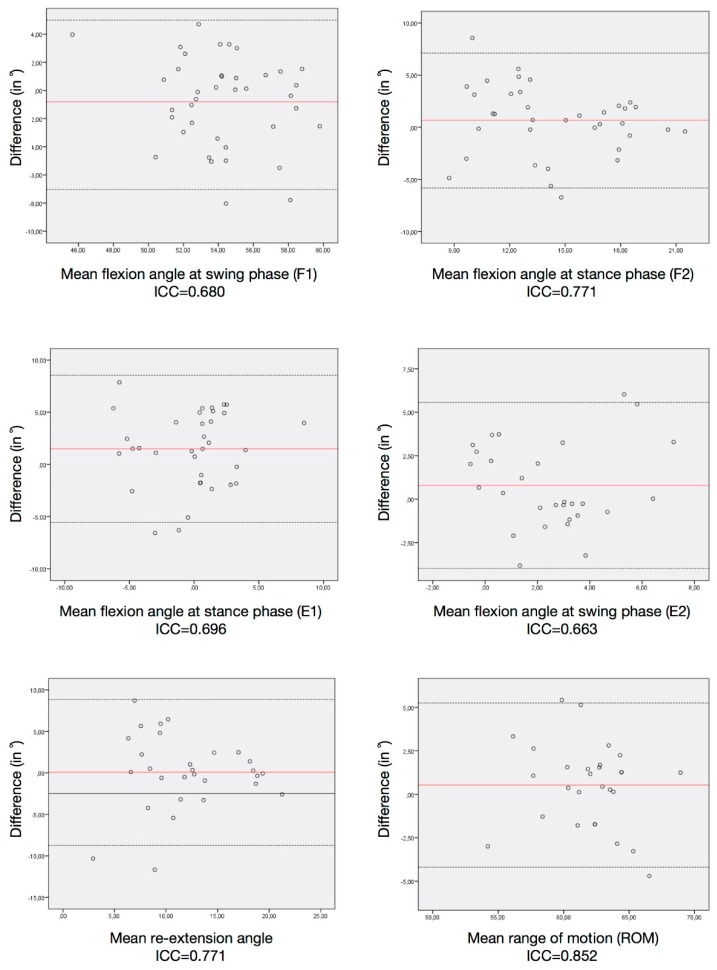
Bland–Altman plots of the discrete parameters from the sensor reliability test. Each graph represents the mean difference (black line) and 1.96× standard deviation of the difference (dashed lines) as recorded by the sensors at two different time points.

The Bland–Altman plots between the two systems are depicted in Figure 4 and the repeatability test in Figure 5 with the corresponding ICC values.

## 4. Discussion

The study showed that during walking, the novel sensors provided sagittal knee kinematic parameters comparable to those obtained with the gold standard of a motion analysis system. 

The interest in easy-to-use and cheap gait analysis systems without local restrictions has increased due to the high cost and difficulty of obtaining kinematic data from individuals using optoelectronic systems in a more artificial setting [20]. Several studies have investigated the performance of novel sensors, comparing them to different measurement methods using disparate devices [11,16,21]. The gold standard method for gait analysis is, however, the camera-based motion detection system [22]. Using a camera system, markers and sensors should be placed separately to determine landmarks, and the data obtained should be analysed for measurement agreement. Although markers for camera systems are often placed according to generally accepted guidelines, there appears to be no consensus for sensor placement or what material to use for placement, partly due to distinctive features of different sensor systems [12]. The placement of sensors on the extremity is therefore a question of interest regarding the inertial sensor units. Several authors have suggested different applications [16,23,24,25]. The data of cutaneous and transosseous inertial sensors on cadaver knees were analysed, and the authors concluded that the cutaneous placement of inertial sensors provides adequate positive correlation with transosseous sensors [25]. Cooper et al. [23] also validated an IMU with a camera motion tracking system connected via a cable, in their paper studying seven subjects. However, although they obtained good agreement between systems, this agreement might be caused by the authors’ placement of camera markers not directly on the extremity but on the sensor materials fixed on the subjects’ legs. Schulze et al. [24] suggested placing sensors on the leg so that they do not constrain knee movements and used kinesiotape for fixation. The authors placed the markers at a distance from the sensors on anatomical landmarks that are easy to identify. To obtain bone proximity and minimal muscle contraction disturbance, the authors suggested the placement of a femoral sensor unit on the lateral distal thigh and of a tibial unit medial to the tibial tuberosity; this method was adopted for the current study. In the current study, markers of the motion analysis system were placed first, followed by the sensor units, which were placed separately from the markers. The sensor units are commonly placed on the subjects using velcro straps, kinesiotape, or bandages [16,23,24,26]. To reduce the fixation method-related disturbances in the sensor recordings and to alleviate any constraint on natural knee movement, self-adhesive bandages were preferred in this study as a practical method.

Furthermore, different sensor systems consist of different numbers of units, with two to seven units per system [11,16,23,24,26,27]. Also, different calibration methods exist for different systems [11,16,24], some of which require squatting of the subject [11]. Seel et al. [10] used gyroscope data for the calibration and calculation of the joint angle; however, this approach can lead to an integration error that accumulates over time. To eliminate the integration error, the fusion of the gyroscope, accelerometer (6-DOF fusion) and magnetic field sensor data (9-DOF fusion) is implemented in each sensor unit. This problem diminishes when using orientation quaternions, since the integration errors are taken care of by the proprietary sensor fusion algorithms. Moreover, the use of the absolute orientation makes the proposed algorithm more suitable for a clinical postoperative setup, since its performance does not depend on the sample rate, and it even works for a set of several static joint positions. The novel device used in this study required only two sensor units, which sufficiently acquired data comparable to the gold standard method. Due to the fact that the calibration process should also be an easy-to-perform task, which is appropriate for postoperative patients, the calibration method required by the developed sensors in this study was more practical compared with previously published methods [11,17,28]. It consisted of arbitrary movements of the lower extremity: simple hip and knee flexion movements and internal and external hip rotations with no minimum or maximum angle limit. Other studies required either squatting from an upright position [11] or up to 80° of knee flexion [17,28], which are difficult to perform by postoperative patients.

Regarding the statistical methods of validation, several studies were performed to reliably detect kinematic parameters for lower extremity joints. Whereas some studies distinctively evaluated more complex and discrete parameters [11,29,30], others only performed RMSE and/or Pearson correlation coefficient analysis on a limited number of participants [24,28]. Tanaka et al. [30] validated a sensor system by comparing it to VICON. The authors only used kinematic waveform data and analysed them using Pearson correlation coefficients on percentage-based gait cycle data without using any discrete parameters. Eltoukhy et al. [29] validated a sensor system analysing spatiotemporal variables, such as step length, step width and stride time on participants walking on a treadmill. They also compared the total range of motion in lower extremity joints; however, no discrete parameters were used for any gait cycle data for any joint. Nuesch et al. [11] compared sensors consisting of seven units with a camera-based system between walking and running on a treadmill. The authors obtained a good agreement between systems by analysing RMSE, multiple correlation coefficients and Bland–Altman plots for the range of motion of lower extremity joints. The analysed data were ROM, specific gait cycle periods and discrete parameters, which were also adopted in the current study. Although this study appears to be the strongest in terms of statistical comparison and agreement evaluation between systems, two major drawbacks were that the measurement was equipment-intensive (the use of a treadmill and the sensor system consisted of seven units) and that the calibration required squatting, which is a difficult task to perform in the clinical setting, especially after knee surgery. The current study, on the other hand, included only two sensor units for practical reasons and overground walking was preferred to treadmill walking.

The results obtained in this study were comparable to, if not better than, the results published in previous similar studies, regarding the RMSE and Pearson correlation coefficients for walking [11,23,24,28]. The RMSE for walking data was 5° in this study, while it was 3° [23,24], 5° [11] and 7° [28] in previously published studies. Regarding discrete parameters, there are no comparable data in the literature, since no previously published study compared peak knee flexion and extension angles in stance or swing phases between different systems. The Bland–Altman plots and related ICC results were comparable to data reported previously by Nuesch et al [11].

Correlation parameters were statistically insignificantly smaller than between-system comparisons when repeated measurements were performed with the sensors. This was easily justified since recordings from both systems belonged to the same gait cycles at a certain time point from the same subject. Repeatability measurements, on the other hand, belonged to different gait cycles of the same subject at different time points. Since gait cycle parameters of the same subject have been shown to differ even within the same day [31,32], these results are reasonable and justified.

Furthermore, the mean E1 values were found to be significantly different, both between systems and within sensors (repeatability). This, however, is of lesser importance in view of the fact that mean E1 values were lower than the standard deviations for this parameter, a result that was also found in a former study [19].

Several authors valued the importance of remote monitoring using sensors on postoperative patients and performed clinical studies [16,21,26,27,33]. Msayib et al. [21] advocated the use of a sensor system on postoperative TKA patients, which allows for remote monitoring of the physiotherapy process. However, the authors neither described the device they used nor validated it using an appropriate method, other than a goniometer measurement. Chiang et al. used sensors clinically on postoperative TKA patients, with no validation of the sensors other than KUKA robot trials [16]. Callies et al. [26], on the other hand, cited a validation study to justify the use of the sensors in the clinical setting of their study. However, their cited research only included correlation coefficient and RMSE calculations to validate the sensors, without benchmarking it against a validated measurement methodology [24]. This research, therefore, constitutes the first study in the literature that is practical, applicable and includes a statistically reliable validation of the sensors. Using only two sensor units with easy-to-use and practical features and an easy-to-perform calibration process, regardless of minor sensor placement differences, the developed sensors were able to detect data comparable to the VICON system measurements. 

This study was not without limitations. It was only performed during walking and with healthy subjects. Future studies should evaluate additional activities, and also include patients with different pathologies.

In conclusion, the sensors developed were shown to provide sagittal knee kinematics comparable to the gold standard of a motion capture system. The data justified the use of these sensor units in further clinical trials as a clinical assessment tool. One of the biggest advantages in using this novel sensor system is the ability to monitor knee functions and mobility in the sagittal plane during the postoperative period without restricting daily life.

## Figures and Tables

**Table 1:** Pearson correlation test, associated *p*-values and RMSE values for kinematic waveforms.

Activity	Correlation Coefficient between Systems (*p*-value)	Correlation Coefficient of Repeatability (*p*-value)	RMSE between Systems	RMSE of Repeatability
Walking	0.96 (0.001)	0.95 (0.001)	5.17°	6.82°

**Table 2:** Flexion and extension of the knee during gait measured by motion capture and the new sensors. (F-Flexion, E-Extension, ROM -range of motion). Bold p-values indicate a nonsignificant difference.

Discrete Parameters from Walking Data	Sensor Mean (SD)	VICON Mean (SD)	*p*-Value
F1	56.1° (8.4°)	53.7° (9.0°)	**0.506**
E1	4.55° (4.28°)	3.35° (3.23°)	0.021
F2	16.5° (6.4°)	15.7° (7.1°)	**0.150**
E2	3.73° (3.89°)	3.64° (2.32°)	**0.751**
Re-extension	13.2° (7.31°)	12.1° (7.65°)	**0.059**
ROM	61.3° (3.85°)	58.2° (4.4°)	**0.776**

**Table 3:** One-sample t-test with the hypothesis that there would be no difference between the mean values recorded at different time points of the same subjects. Bold p-values indicate a nonsignificant difference.

Discrete Parameters from Walking Data	Sensor First Recording Mean (SD)	Sensor Second Recording Mean (SD)	*p*-Value
F1	53.80° (2.9°)	54.6° (3.5°)	**0.122**
E1	0.58° (3.8°)	−0.91° (3.6°)	0.019
F2	14.7° (3.5)	14.0° (4.1)	**0.235**
E2	2.9° (2.4)	2.1° (2.4)	**0.092**
Re-extension	12.0° (5.2)	11.9° (5.2)	**0.939**
ROM	62.2° (3.17)	61.6° (3.56)	**0.254**

**Table 4:** Intra-class correlation coefficients (ICCs) for discrete parameters during walking between the systems with a 95% limit of agreement.

Discrete Parameters from Walking Data	ICC
F1	0.923
E1	0.542
F2	0.859
E2	0.684
Re-extension	0.829
ROM	0.701

**Table 5:** ICCs for discrete parameters during walking within the sensor recordings with a 95% limit of agreement.

Discrete Parameters from Walking Data	ICC
F1	0.680
E1	0.696
F2	0.771
E2	0.663
Re-extension	0.771
ROM	0.852
